# Computational approaches to alleviate alarm fatigue in intensive care medicine: A systematic literature review

**DOI:** 10.3389/fdgth.2022.843747

**Published:** 2022-08-16

**Authors:** Jonas Chromik, Sophie Anne Ines Klopfenstein, Bjarne Pfitzner, Zeena-Carola Sinno, Bert Arnrich, Felix Balzer, Akira-Sebastian Poncette

**Affiliations:** ^1^ Digital Health – Connected Healthcare, Hasso Plattner Institute, University of Potsdam, Rudolf-Breitscheid-Straße 187, Potsdam, Germany; ^2^ Charité – Universitätsmedizin Berlin, corporate member of Freie Universität Berlin and Humboldt–Universität zu Berlin, Institute of Medical Informatics, Charitéplatz 1, Berlin, Germany; ^3^ Berlin Institute of Health at Charité – Universitätsmedizin Berlin, Core Facility Digital Medicine and Interoperability, Charitéplatz 1,Berlin, Germany; ^4^ Charité – Universitätsmedizin Berlin, corporate member of Freie Universität Berlin and Humboldt-Universität zu Berlin, Department of Anesthesiology and Intensive Care Medicine, Charitéplatz 1, Berlin, Germany

**Keywords:** Alarm fatigue, alarm management, alarm optimisation, intensive care unit, IT system, patient monitoring, ICU, critical care

## Abstract

Patient monitoring technology has been used to guide therapy and alert staff when a vital sign leaves a predefined range in the intensive care unit (ICU) for decades. However, large amounts of technically false or clinically irrelevant alarms provoke alarm fatigue in staff leading to desensitisation towards critical alarms. With this systematic review, we are following the Preferred Reporting Items for Systematic Reviews (PRISMA) checklist in order to summarise scientific efforts that aimed to develop IT systems to reduce alarm fatigue in ICUs. 69 peer-reviewed publications were included. The majority of publications targeted the avoidance of technically false alarms, while the remainder focused on prediction of patient deterioration or alarm presentation. The investigated alarm types were mostly associated with heart rate or arrhythmia, followed by arterial blood pressure, oxygen saturation, and respiratory rate. Most publications focused on the development of software solutions, some on wearables, smartphones, or headmounted displays for delivering alarms to staff. The most commonly used statistical models were tree-based. In conclusion, we found strong evidence that alarm fatigue can be alleviated by IT-based solutions. However, future efforts should focus more on the avoidance of clinically non-actionable alarms which could be accelerated by improving the data availability.

**Systematic Review Registration:** https://www.crd.york.ac.uk/prospero/display_record.php?ID=CRD42021233461, identifier: CRD42021233461.

## 1. Introduction

In an intensive care unit (ICU), many different sensors automatically monitor vital signs (e.g. heart rate (HR), arterial blood pressure (ABP), and oxygen saturation (SpO_2_)), leading to improved patient safety. Alarms occur for example when a vital sign exceeds or drops below a predefined threshold.

Of all patient monitoring alarms, 72% to 99% are either technically false (e.g. due to an artefact) or clinically irrelevant, i.e. technically true but not actionable (1). Hence not every alarm indicates a change in the patient’s medical situation or requires a medical intervention (2).

Alarm fatigue occurs as a result of an overwhelming amount of alarms. This sensory overload can lead to alarm desensitisation and a loss of competence of the ICU staff (physicians and nurses) in dealing with alarms, ultimately resulting in patient harm or even death (3–5). ICU staff experiencing alarm fatigue might delay or dismiss alarms, wrongly adjust alarm thresholds, or struggle to evaluate and prioritise alarms (1). In both, patients and staff, excessive amounts of alarms can cause stress, leading to distraction from work and disrupted circadian rhythm (6–9).

The problem of alarm fatigue has been widely investigated, both qualitatively (10) and quantitatively (11). Many different approaches to alleviate alarm fatigue have already been evaluated and tested: For example, workshops and trainings for ICU staff, implementation of guidelines for alarm management, adjustments of threshold, and development of algorithms (12, 13). There is, however, no ultimate and generally applicable solution yet. Excessive amounts of alarms can be caused by many potentially interacting factors. As of now, there is no standardised tool to measure alarm burden. Alarm fatigue does not only involve individuals but entire organisations (14) and the roots of the problem are very diverse. Solely reducing the number of alarms might not resolve the problem of alarm fatigue or change the attitude of ICU staff towards the alarm situation (15). Patient monitoring alarms are triggered through an IT system that uses signals produced by various sensors. However, currently used IT systems lack positive predictive value in the alarm generation, subsequently causing large amounts of false-positive alarms. We presume that a more holistic IT-based approach might be a promising approach to alleviate alarm fatigue in intensive care medicine.

With this systematic literature review, we address the following research questions: (1) What are IT-based approaches to improve alarm management in intensive-care medicine? And (2) how do these IT systems contribute to alarm management in intensive-care medicine?

In terms of PICOS, we specify this review as follows:

• The *problem* is alarm fatigue in intensive-care medicine, i.e. intensive care unit (ICU), intermediate care unit (IMC), neonatal intensive care unit (NICU), and post-anesthesia care unit (PACU).
• The *interventions* are novel IT systems dealing with medical alarms influencing them in any kind, for example by alarm prediction, novel methods of alarm presentation, alarm prioritisation, alarm suppression, alarm delay, or personal alarming.
• This is *compared with* the state-of-the-art ICU setup without the respective intervention.
• The *outcome of interest* is reduction in alarm burden, through reduction of the number of alarms per staff member or reduction of noise due to alarms.
• And there are no restrictions on *study type* or time frame.

In this systematic literature review, we closely follow the PRISMA process (16). The structure of this work closely resembles the PRISMA checklist and deviates only when comprehensibility and conciseness would otherwise suffer.

## 2. Methods

### 2.1. Eligibility criteria

In addition to the PICOS defined in Section 1, we applied the following exclusion criteria to the literature:

• publications that are not written in English language
• publications that do not provide a full text
• publications that are not peer-reviewed.

### 2.2. Information sources

As primary source of information, we employed the five public literature databases: ACM Digital Library^1^, arXiv^2^, IEEE Xplore^3^, PubMed^4^, Web of Science^5^. These databases were queried as described in Section 2.3 to produce a core set of literature which was subsequently augmented by additional literature.

### 2.3. Search strategy

**Figure 1:**
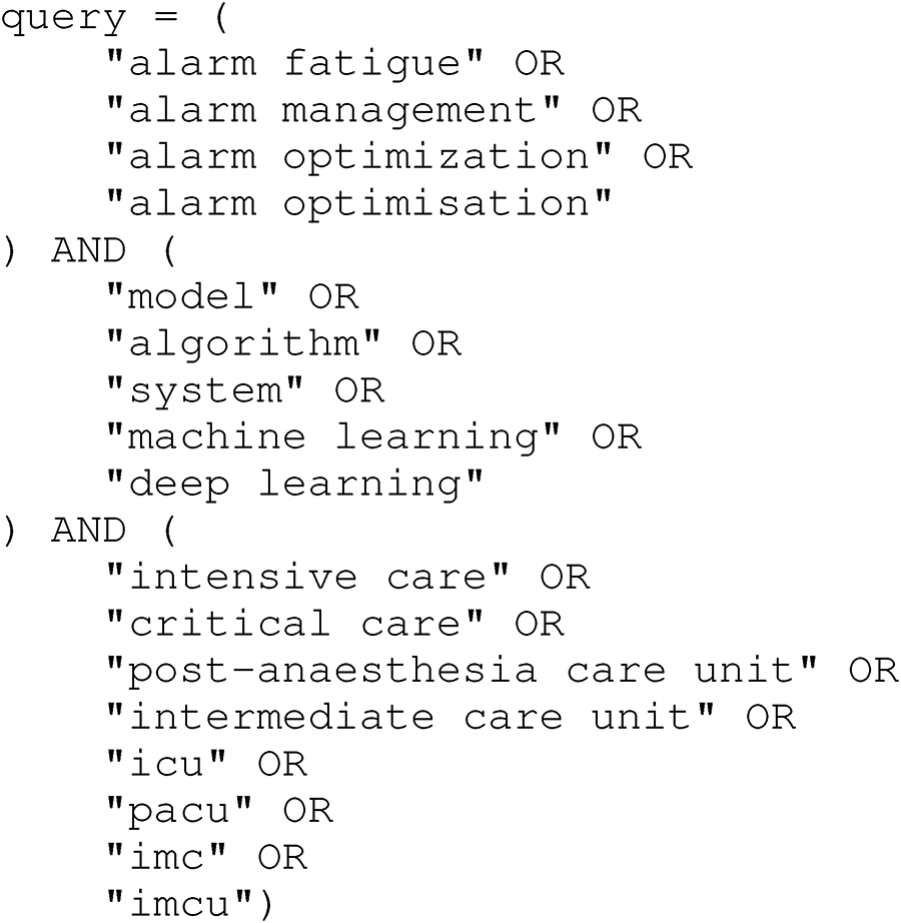
Database query used on all public databases. The first part of the query sets the topic domain, the seconds part defines the computer science aspects, and the third part defines the medical aspects.

**Figure 2:**
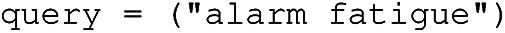
Simplified query that was used additionally on IEEE Xplore.

All databases listed in Section 2.2 were queried as defined in Figure 1. Additionally, IEEE Xplore was queried again as defined in Figure 2. All public database queries were executed on December 4, 2020. All fields were queried without any further restrictions.

Apart from querying public databases, we also employed an internal literature database as a source for additional literature. Furthermore, we noticed that many publications describe solutions to the PhysioNet/CinC Challenge 2015^6^ (17) which is why we included papers related to this challenge^7^ as additional literature.

### 2.4. Study selection

We have to ensure that the publications we found conform with both the topic of this literature review defined in Section 1 and the additional formal eligibility criteria defined in Section 2.1. Therefore, we screened title, abstract, and keywords of each publication found before assessing the full-text version and checked if one of the exclusion criteria listed below is met. If at least one criterion was met, we excluded the publication from the subsequent in-depth review process. There were cases where we could not definitely decide upon the exclusion criteria by title, abstract, and keywords alone. In these cases, we retained the publication in questions to be potentially excluded in a later stage of the review. The exclusion criteria are designed to represent PICOS as well as eligibility criteria:

• Does not concern (an) *implemented IT solution*(s)
• Does not concern *intensive-care medicine*
• Does not concern *alarm management*
• No full text can be found (e.g. abstracts of talks)
• Record is assumed to be not peer-reviewed

### 2.5. Data collection process

For each paper, we extracted the author names, the title, and the year of publication as metadata. Furthermore, we answer the questions listed in Section 2.2 based on the respective publication. Each publication was initially processed by one of the authors of this review. If one or more data items could not confidently be extracted by the responsible reviewer, a second reviewer was assigned to help clarify the issue.

### 2.6. Data items

From each paper, we extracted where possible following data items formulated as questions including sample answers.

#### 2.6.1. Objective

What is the core objective of the publication? Exemplary objectives are the reduction in false alarms or forecasting of alarm to reduce the urgency that is usually associated with an alarm. This data item asks for the contribution of the publication from a medical perspective.

#### 2.6.2. Approach

How is the core objective achieved? Exemplary approaches are the use of machine learning models or novel methods of alarm presentation. This data item asks for the technical implementation from a computer science perspective.

#### 2.6.3. Alarm types

Which alarms are targeted? Examples are alarms due to tachycardia or low blood pressure.

#### 2.6.4. Hardware involvement

Does the presented system involve specific hardware that is not a usual part of an ICU setup? If so: Which kind of hardware was used? One example would be smartphones for additional data collection.

#### 2.6.5. Data

Which data sets are used by the system? Predominantly, we were interested in whether databases were used or if data collection was part of the research leading to the publication. If databases were used, we were interested in which database was used, e.g. MIMIC-II (18).

#### 2.6.6. Models

If there are predictive models in use: Which model types are used? Examples are support vector machines (SVMs) or neural networks (NNs).

#### 2.6.7. Patients

Are there specific patient characteristics? If yes: Which? Examples are limitations of specific age groups or patients having specific diseases.

#### 2.6.8. User study

If there was a user study of some kind: How many participants were involved?

### 2.7. Risk of bias in individual studies

We expect a risk of bias in the individual publications. We focused on engineering approaches for alarm management and expected to find many feasibility studies among the publications. Feasibility studies aim to show that the proposed solution works and how it works. We expect a bias favouring proposed solutions in feasibility studies because the primary objective of a feasibility study is not to rigorously compare the proposed solution to an alternative by means of a controlled experiment such as a randomised controlled trial (RCT). The authors of such feasibility studies may be biased as the creators of the solutions themselves.

### 2.8. Summary measures

To the best of the authors’ knowledge, there is no standard measure of alarm fatigue available at the time of submission of this review. Also, we did not find any such measure while conducting the literature search. Therefore, we can not provide a quantitative summary measure for the proposed solutions covered by this review. We do, however, provide statistics on the data items when this is feasible to show how alarm fatigue was tried to be alleviated in the past.

### 2.9. Synthesis of results

We analysed each data item separately for all publications considered in this review and summarise our findings specific to each item in Section 3.2. As an additional analysis, we combined publications with similarities by grouping them together. Grouping can be done by each of the data items defined in Section 2.6 as long as there are striking similarities. Consequently, a publication can be part of multiple groups. Findings for these subgroups are to be found in Section 3.3.

### 2.10. Risk of bias across studies

We split the publications among the authors. This introduces a bias from the different professional background of the authors, either computer science or medicine. Further, there is a positive publication bias prevalent that makes the publication of negative findings less likely (19, 20). Applied to this review, we have to assume that the probability of publication is lower for solutions that were tried but did not contribute to the alleviation of alarm fatigue.

## 3. Results

### 3.1. Study selection

We queried the public literature databases listed in Section 2.2 using the query stated in Figure 1 and found *184 records* (including duplicates). Table 1 shows the number of records yielded by each database as well as how many records we found in multiple databases. We treat publications that we found in more than one database as duplicates that we merge in the duplicate removal stage. We found many duplicates across PubMed and Web of Science since both databases aggregate publications from multiple publishers. In contrast, ACM and IEEE are publishers of their own and only list publication that they published. Consequently, there is no overlap between these databases. After duplicate removal, we retain *135 records* from public databases.

**Table 1:** Matrix of duplicates across different literature databases. The numbers state how many publications we found in both literature databases (row and column).

	ACM Digital Library	arXiv	IEEE Xplore	PubMed	Web of Science
ACM Digital Library	25	0	0	0	1
arXiv	0	0	0	0	0
IEEE Xplore	0	0	14	4	11
PubMed	0	0	4	84	33
Web of Science	1	0	11	33	61

Additionally, we employed other sources as described in Section 2.3. The additional number of records yielded by these other sources is shown in Table 2. After adding these *43 records*, we have *178 records* in total to be screened.

**Table 2:** Additional records yielded by other sources.

Database	No. of Records
Referenced in included papers	+1
IEEE Xplore with “alarm fatigue” query	+8
Internal literature database	+16
PhysioNet/CinC Challenge 2015	+18

**Figure 3:**
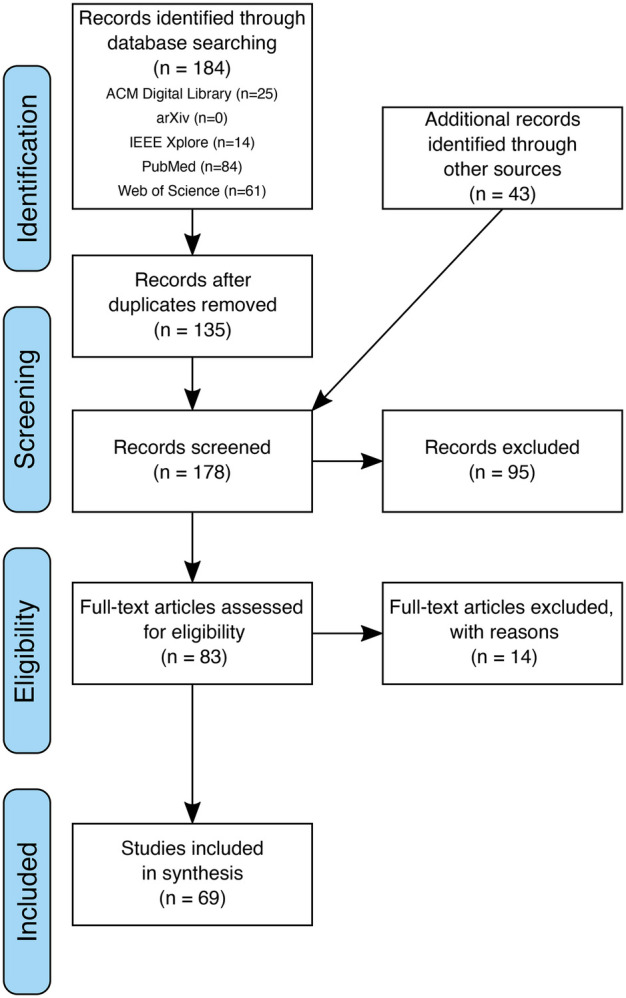
PRISMA Flowchart.

Title, abstract, and keywords of these 178 records were screened according to the eligibility criteria defined in Section 2.4. After screening, we excluded *95 records*, hence retaining *83 records* for full-text access. During the screening, we decided to retain records where eligibility was unclear leaving us with the option to exclude these records later on after full-text access. The *14 records* excluded after full-text access (with reason) are listed in Table 3. After this final round of exclusion, *69 records* were left to be included in the synthesis. The complete process of record identification, screening, eligibility assessment with all removals and exclusions during the process, is depicted in a prototypical PRISMA flowchart in Figure 3.

**Table 3:** Records excluded after full-text access (with reason).

Reason	Records	Count
No IT solution	(21–31)	11
Not alarm management	(32–34)	3

**Figure 4:**
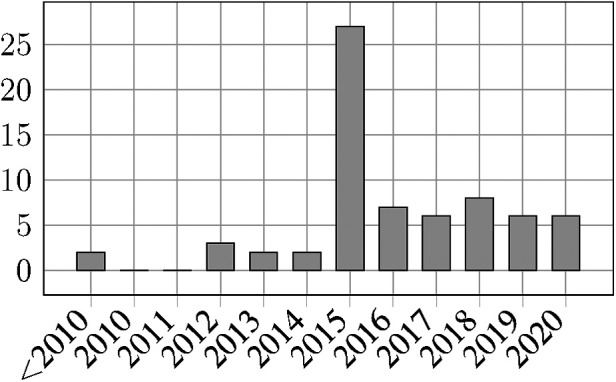
Number of included publications per year.

Figure 4 shows the distribution of the reviewed publication throughout the years. There is a very noticeable peak in 2015 which can be attributed to the PhysioNet/Computing in Cardiology challenge of this year. Also, the number of publications per year remains at an increased level throughout the subsequent years. This can be attributed to publications that used the challenge dataset but were only published after the challenge has ended. In Section 1 we describe the impact of this challenge in greater detail.

### 3.2. Synthesis of data items

#### 3.2.1. Objective and approach

After data extraction, we chose to consider objective and approach together. This is reasonable since the distinction between means and ends is not always clear. As a result of the inclusion and exclusion criteria (see Section 2), all publications have in common that counteracting alarm fatigue is part of their objectives. However, the concrete way of how this is achieved varies.

We identified three major approaches utilised in order to counteract alarm fatigue:

##### 3.2.1.1. Reduction of technically false alarms

Schmid et al. (35) assigns to alarm the two properties of technical correctness and clinical relevance. Consequently, there are (1) technically false alarms where the cause of the alarm does not correspond to reality, (2) technically correct but clinically irrelevant (i.e. non-actionable) alarms where the cause of the alarm is rooted in reality but there is no consequence to the alarm, and (3) technically correct and clinically relevant (i.e. actionable) alarms.

We used the terminology from (35) to classify the publications we reviewed. Among the publications that aim at the numerical reduction of alarms, the majority of publications address only technical correctness and not clinical relevance. This is prominently the case in the “avoidance of false arrhythmia alarms” subgroup which we identified and describe in Section 1.

One way in which clinical relevance is addressed is via *SuperAlarm* patterns (see Section 3.3.4). However, the aim of these works is not to avoid clinically false alarms but to identify specific highly clinically relevant combinations of alarms.

##### 3.2.1.2. Per-patient risk assessment

In the reviewed literature we found an overlap with the related research topic of clinical risk models (e.g. prediction of mortality, post-operative complications, and patient deterioration in general). While clinical risk models do not aim at the alleviation of alarm fatigue as their primary objective, they can still be utilised in order to prioritise alarms. Jo et al. (36) states that “High accuracy in mortality prediction helps nurses manage patient care by placing patients in different priority queues. It also enhances nurses’ efficiency by reducing the number of false alarms, which cause them alarm fatigue and desensiti[s]e them to real alarms.” Publications with this objective are described in the “prediction of patient deterioration” subgroup (Section 5).

##### 3.2.1.3. Changes in the ways alarms are presented

Besides avoiding false alarms and prioritising true alarms, there is also research in the field of human-computer interaction aiming at changing the way alarms are presented. Most of these works focus on reducing the acoustic load in ICUs. This is described in greater detail in Section 3.

#### 3.2.2. Alarm types

Among the 69 reviewed publications, we identified two principal groups with respect to the targeted alarm types:

1. 56 publications presenting *alarm-specific* solutions that target a predefined set of alarms.
2. 13 publications presenting *alarm-agnostic* solutions, which do not specify any type of alarms or did not use alarms to be developed.

As many publications do not mention the sensor(s) used to measure the vital signs or parameters leading to those alarms, we decided to create clusters for the parameters triggering the alarms.

**Figure 5:**
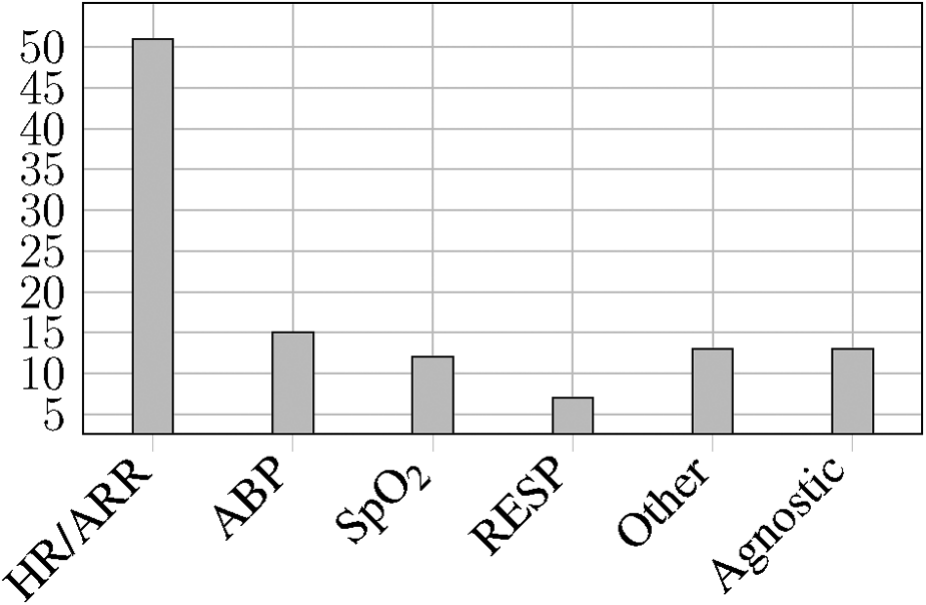
Investigated alarm types or clusters.

Figure 5 shows the frequency distribution of examined alarm types and/or clusters.

##### 3.2.2.1. Alarm-specific solutions

56 reviewed publications investigated one or several types of alarms. We identified the following alarm types and/or clusters:

• HR/ARR: Heart rate related alarms triggered by a pulse oximeter or electrocardiogram (ECG), in case of pulse and/or heart rate threshold violation (both terms were often used interchangeably in the reviewed publications) or triggered when arrhythmia (ARR) patterns like asystole (ASY), extreme bradycardia (EBC), extreme tachycardia (ETC), ventricular fibrillation (VFib), ventricular flutter (VFl), or ventricular tachycardia (VT) were recognised (n=51).
• ABP: Arterial blood pressure alarms with blood pressure being measured non-invasively and/or invasively (n=15).
• SpO_2_: Oxygen saturation alarms as per pulse oximeter (n=12).
• RESP: Respiration-related alarms from ECG/impedance pneumography or ventilator triggered by respiratory rate, apnea, and further ventilation parameters (n=7).
• Other: Alarms triggered by further, less frequent parameters namely intracranial pressure (ICP) (n=4), central venous pressure (n=3), body temperature (n=2), perfusion (n=2), and pulmonary arterial pressure (n=2).

We analysed the number of alarm types and/or clusters evaluated by the reviewed publications. We noticed that most of the publications focused on only one alarm type or cluster (n=39) and if so, most frequently on the HR/ARR cluster. The described solutions can be uni- or multimodal, hence using different data sources such as laboratory values as additional information.

##### 3.2.2.2. Alarm-agnostic solutions

13 reviewed publications do not specify the alarm types they are focusing on (n=11) or do not use alarms to develop a solution aiming to alleviate alarm fatigue (n=2). Hence, these solutions claim to be applicable to all types of alarm without or with only minor adaptations.

Wilken et al. (37) presents a system enabling healthcare workers to analyse the alarm situation on their ICU using a data warehouse for daily clinical decision making and further research. The system does not target any specific set of alarms.

5 publications from Cobus et al. present devices to counteract acoustic load, reduce high cognitive load, and the risk of alarm fatigue in ICUs. Their prototypes were not restricted to any alarm types, hence all alarms are possibly targeted. These publications are further described in Section 3.3.3.

5 publications proposed the use of *SuperAlarm* patterns predicting code blue events and using all types of alarms as input. They are further described in Section 3.3.4.

2 publications do not use any alarm data to develop solutions to reduce alarm burden by predicting clinical deterioration (38) or mortality along with a de-prioritisation of alarms from patients with a lower mortality probability (36) (see Section 3.3.5).

#### 3.2.3. Hardware involvement

We found only a few publications proposing an alarm management system that involves hardware. Mainly, systems rely on existing hardware, most prominently patient monitors. Data collected through patient monitors is captured in databases such as MIMIC-II (18) (see Section 3.2.4). Solely software-based systems use these databases for evaluation and – if machine learning is involved – training of models.

As an exception to this, there is hardware involved in the subgroup “novel means of alarm presentation” (see Section 3.3.3). The publications of Cobus et al. present and utilise devices like a vibrotactile wearable alarmsystem or head-mounted display for delivering alarm to the medical staff. Further, there is a system called “D.A.S.H.” Greer et al. (39) which adapts the volume of alarms to the background noise to avoid unnecessary noise pollution. This is implemented in hardware as a physical system using “an Arduino Mega microcontroller board with [a] digital and analog[ue] input/output pins for computation purposes, a Wave Shield for added sound quality, microphones for sound acquisition, a pulse sensor for measuring patient HR, and a loudspeaker for sound output.”

Additionally, smartphones are commonly used as a hardware component for two distinct purposes. Firstly, smartphones are used as low-cost and ubiquitously available sensing hardware, e.g. in (40). Secondly, smartphones can be used as a communication device to facilitate personal alarming, notifying a specific member of staff instead of informing all members of staff in the vicinity of the problem with a broadcast-style audible alarm. This is by way of example demonstrated by (41).

Finally, there is specific simulation hardware that is used in order to better understand alarm fatigue. Kobayashi (42) uses manikins (i.e. SimMan 2G and an ECG-enabled Resusci-Anne by Laerdal) to build a simulation space for studying alarm fatigue.

#### 3.2.4. Data

The vast majority of publications reviewed in this work use custom data sets for training and/or evaluating their proposed IT systems. Custom data set in this case either means a data set specifically created for this publication or a data set that we could not trace back to another publication.

5 publications use no data at all or do not mention the use of data. This is the case in (43–47).

Furthermore, the MIMIC-II database is an important material in alarm fatigue research. MIMIC-II is used by (36, 48–53). It is, however, noteworthy that specifically MIMIC-II is used while its successor MIMIC-III (54) and other ICU databases such as HiRID (55) and eICU CRD (56) are not even mentioned in the publications under review. A reason for this might be that MIMIC-III, HiRID, and eICU CRD are relatively new while MIMIC-II is publicly available since 2010 (18). This manifests a need for further research as it needs to be evaluated how the proposed IT solutions can benefit from more recent databases.

Another important data source for alarm fatigue research is PhysioNet (57). PhysioNet both helps to distribute ICU databases like the ones mentioned above and hosts yearly challenges in conjunction with the Computing in Cardiology conference. These challenges include data sets that are used as part of the challenge but also for subsequent research. In the publications we reviewed (40, 58) mention the use of a data set distributed via PhysioNet but do not offer any specifics, (59) uses the data from the 2009’s and the 2014’s challenge, and (60) uses the data from the 2011’s challenge.

We want to put special focus on the data set associated with the 2015’s challenge. This data set contains physiological signals preceding arrhythmia alarms which is extremely relevant in terms of alarm fatigue especially concerning the improvement of technical correctness of alarms (35). Besides the submission to the challenge described in Section 3.3.1, the following publications are also using this data set: (61–68)

#### 3.2.5. Models

Out of the papers that utilise models in some way, the most commonly used ones are tree-based models (decision tree (DT), random forest (RF), extremely randomised trees (ERT), gradient boosting machine (GBM)). As so-called *explainable models* they have the benefit of producing humanly-comprehensible reasons for their decisions. Further applied explainable models are logistic regression (LR), fuzzy logic and case-based reasoning (CBR). Apart from these, the most frequent models are SVMs. Deep networks such as NNs and long-short term memorys (LSTMs) are less popular in the reviewed literature, which could be due to the requirement of large databases or their black-box character which is not that suitable for the medical field. 10 papers describe other models, such as clustering- or threshold-based models, or rare regression models.

#### 3.2.6. Patients

Most studies utilised monitoring data produced by adult patients admitted to ICUs in the United States or Europe. Several studies relied on data from the PhysioNet databases such as MIMIC (see Section 3.2.4). However, some publications included own data especially regarding neonatal or paediatric (41, 69), cardiothoracic (70, 71) or neurosurgical (72–74) patients. The majority of data included came from critically ill patients in the ICU, a minority from patients in IMCs (75).

#### 3.2.7. User study

A user study is performed in eight of the reviewed papers. “User study” was defined as an evaluation of the usability or an assessment of the proposed method of alarm reduction. Most of these papers are from Cobus et al. since their proposed method includes hardware. These user studies consisted of the testing of the prototype (47, 73, 76, 77), performance of tasks (46, 47, 77), interviews (45, 58), as well as pilot sessions (42). Not all the papers included the number or the background of the participants in their user study. Of those who provided this information, the number of participants ranged from 9–15, all of them healthcare workers. Table 4 shows a complete list of user studies found during this review as well as their characteristics.

**Table 4:** Papers with a user study.

Publication	Type of user study	# of participants	Participant background
(73)	Performance of tasks	11	not specified
(76)	Assessment of the prototype	12	Nurses
(47)	Performance of tasks, assessment of the prototype	12	Nurses
(77)	Performance of tasks, assessment of the prototype.	15	not specified
(45)	Semi structured interviews (before development of the prototype)	9	Healthcare workers
(58)	Interviews	not specified	not specified
(42)	Two pilot sessions	not specified	not specified

### 3.3. Subgroup analyses

Among the reviewed papers, we identified five subgroups with publications having common themes in terms of objective or approach. These subgroups are partially overlapping meaning that one paper can be part of more than one subgroup for example when false arrhythmia alarms (Section 3.3.1) are avoided using signal quality indicators (SQIs) (Section 3.3.6). In the following, we present the identified subgroups.

#### 3.3.1. Avoidance of false arrhythmia alarms

This subgroup contains the 20 additional publications identified through the PhysioNet/CinC Challenge 2015, namely (17, 62, 78–95). Furthermore, we identified 8 more publications that also belong to this subgroup, namely (40, 49, 61, 64–66, 68). These publications share the common objective of alleviating alarm fatigue through the reduction of false arrhythmia alarms.

For the publications from the PhysioNet/CinC Challenge 2015, (17) is the overview paper which describes the challenge and also compares the different solutions to the challenge. All solutions use the same challenge data set of 1250 segments (750 for training and 500 for testing after submission). Each segment is 5:00 minutes for real-time and 5:30 minutes for retrospective solutions. The segments include ABP, ECG, photoplethysmogram (PPG), and respiration (RESP) waveform signals. However, not all solution to the challenge use all signals. Alarms targeted by this challenge are ASY, EBC, ETC, VT, and VFib or VFl (VFib and VFl as one alarm). All solutions are solely software-based and do not involve hardware. While some solutions use hand-crafted algorithms others use predictive models i.e. DTs, RFs, SVMs, and NNs. There was no user study since the algorithms were evaluated against a data set.

The publications in this subgroup that did not directly emerge from the PhysioNet / CinC Challenge 2015 were all published in the same year or the years following the challenge. Eerikäinen et al. (66) seems to be a follow-up paper to (81) using the same data set and a similar algorithmic approach. Yanar and Dogrusoz (64) also provides a solution to the PhysioNet / CinC Challenge 2015 but was published only in 2017 and solely focuses on ASY and EBC alarms while also using the challenge data set. Yanar and Dogrusoz (65) seems to be a follow-up paper specifically focusing on VFib and VFl alarms and also using the challenge data set.

Also using the PhysioNet/CinC Challenge 2015 dataset, Gajowniczek et al. (68) proposes the use of weighted RFs for arrhythmia detection. Targeting the full set of available arrhythmia alarms, the authors show that weighted RFs show the best results in terms of area under the receiver operating curve and challenge score.

Puri et al. (40) presents iCarMa, an inexpensive smartphone-based system for cardiac monitoring using PPG sensors which is able to detect ASY, EBC, ETC, VFl, and VT. An important aspect of this work in terms of alarm fatigue alleviation is the strong focus on signal decorruption and avoidance of technically false alarms. The findings for PPG signals can be used to facilitate ECG-based arrhythmia detection in patient monitors.

Roychoudhury et al. (50), Wang et al. (49) and Srivastava et al. (61) use data from the MIMIC-II database (18) for detecting false arrhythmia alarms. Roychoudhury et al. (50) developed a method for extracting shapelets from the ECG signal that are distinctive for false alarms concerning ASY and VT that can be matched for prediction while considering some uncertainty. The other two publications present classical machine learning (ML) systems. While (49) uses only ABP and ECG signals, Srivastava et al. (61) additionally uses a PPG signal. Both systems try to detect false alarms concerning ASY, EBC, ETC, VT, and VFib. To this end, (49) uses SVM, DT, and k nearest neighbours (KNN) models and (61) uses a RF classifier in conjunction with SQIs.

#### 3.3.2. Avoidance of false intracranial pressure alarms

This rather small subgroup includes only two publications, namely (73, 96). Both papers have two authors in common and are based on the same dataset, which was collected retrospectively from a NeuroICU at the University of California, Los Angeles Medical Center between March 2010 and October 2012. The dataset consists of 8000 alarms (which is a subset of the collected alarms, reduced for convenience purposes), 4791 of which were manually labelled. Continuous ICP signals were also extracted from the monitors. Both papers explore methods based on pattern recognition to improve the detection accuracy of ICP alarms by predicting the morphology of an ICP wave before an alarm occurs.

In the first paper, the authors introduce three supervised regression models using only the labelled dataset: Spectral regression, kernel spectral regression, and SVMs. They also compare conditional distribution for feature encoding with morphological clustering and analysis of ICP pulse (MOCAIP) tracking used to extract segments from ICP waves. In the second paper, the authors explore the use of semi-supervised learning models to test the detection accuracy of ICP alarms using a smaller set of labelled data. They compare two models: Kernel spectral regression and SVM, both adjusted to be used in a semi-supervised manner.

#### 3.3.3. Novel means of alarm presentation

This subgroup consists of (39, 44–47, 76, 77). The first publication presents a dynamic system for alarm loudness regulation with respect to ambient noise (39). The second publication proposes a distributed, secure system allowing healthcare workers to get personalised alarm notifications. The system is based on a service-oriented device connectivity standard family, namely IEEE 11073, ensuring the interoperability of devices from different producers, and allowing the system to be extended and further developed in the future (44). The latter five publications (all by the same first author) describe devices using multimodal concepts to forward and present alarms acoustically and non-acoustically to the responsible caregiver. The modalities used are vibration, peripheral light and (bone conduction) sound. They present a vibrotactile wearable alarm system worn on the upper arm (47) as well as different versions of a head-mounted display for alarm presentation showing an evolution throughout the years 2017 to 2019 (45, 46, 76, 77). The head-mounted display delivers alarms as well as alarm-relevant information, such as the patient’s vital data, the sensor, the respective patient’s name or its priority. The authors aimed at reducing the high cognitive load, “counteract[ing] the acoustic load on intensive care units,” “convey[ing] alarms directly to the responsible nurse,” ultimately leading to patient safety improvement and counteracting of alarm fatigue. All publications described in this section presented their devices with the help of prototypes/demonstrators, and some were tested in small user studies (see Section 3.2.7).

#### 3.3.4. SuperAlarm patterns

One research group published a number of papers revolving around the same concept of *SuperAlarm* patterns (97–101). These are combinations of alarms preceding a code blue event on ICU. Utilising the Apriori algorithm (102), appropriate candidate *SuperAlarm* patterns are mined from the training data. They are then filtered using a support threshold required amongst code blue training samples, closed itemset mining, as well as a threshold for the false positive rate (FPR) as indicated by negative (non-code blue) training samples. Based on the final *SuperAlarm* pattern set, time windows before a new alarm are analysed and classified as valid or invalid. (98) In extensions to that work, laboratory test results are included in the *SuperAlarm* patterns (100), or a so-called “weighted accumulated occurrence representation (WAOR)” also encodes the temporal information of preceding alarms, allowing for more sophisticated classification with LR (99). The latter approach has also shown good results in cross-institutional application (97). Finally, the same research group tried training a LSTM on the raw alarm information without extracting patterns which yielded comparable results (101).

#### 3.3.5. Prediction of patient deterioration

This subgroup includes (36, 38, 48, 53, 103). These publications describe approaches for predicting patient deterioration, which can reduce alarm fatigue by scheduling an action in the near future, preempting further critical alarms. Conversely, alarms for patients with a low predicted chance for deterioration could be suppressed. Since this is a large research field on its own, we only include papers specifically mentioning alarm fatigue.

First, Bhattacharya et al. (48) investigate the prediction of an acute hypotensive episode (AHE) up to 120 minutes before its start. The used data are mean arterial pressure (MAP) measurements ($\frac{2}{3}\cdot$ systolic ABP $-\frac{1}{3}\cdot$ diastolic ABP) from the MIMIC-II database (18), measured every minute. Depending on MAP means and standard deviations within the patients in the training set, two decision boundaries are fitted to classify between risk and no risk of an AHE. MAP means between the decision boundaries are treated separately by determining the mean squared deviation between the sample mean and all means in the two classes, assigning the class label according to the lower deviation. Similarly, Shin et al. (53) predict sustained hypotensive episodes (SHEs), defined as the aforementioned AHEs, for patients receiving vasopressor infusion. They train a LR and an auto-regressive model, and include vasopressor dose information in addition to the MAP values. However, they only achieve a good prediction between two and ten minutes before the SHE.

Jo et al. (36) details a unique approach utilising state transition models based on textual information from nursing notes. The models are used to predict 1-day, 1-week, 1-month, 6-month and 1-year mortality of the patient.

Hu et al. (38) use a NN with a single hidden layer to predict ICU transfer or cardiac arrest for patients four to eight hours in advance. The model is given laboratory data and mean vital sign values and produces a binary output.

Looking at a much shorter time frame, Joshi et al. (103) use data from the NICU to predict critical alarms occurring one to three minutes after a warning produced by HR, SpO_2_ or RESP surpassing a pre-defined threshold. This goal is achieved by training a GBM based on DTs with Gini impurity and max depth of 6 with a large feature set comprised of patient information and features extracted from continuous vital parameter measurements leading up to the warning.

#### 3.3.6. Usage of SQIs for avoidance of false alarms

According to our review, SQIs are a popular tool in the attempt to alleviate alarm fatigue by reducing the amount of technically false alarms (as opposed to technically correct but clinically irrelevant i.e. non-actionable alarms, see (35)). Especially the correctness of heart-related alarms is often assessed via SQIs. This is why there is a considerable intersection between this subgroup and the “avoidance of false arrhythmia alarms” subgroup (see Section 3.3.1). Specifically, (78–82, 84, 85, 88, 94) are using SQIs and are part of the PhysioNet / CinC Challenge 2015; (61, 66) are using SQIs and are also part of the arrhythmia subgroup although not directly associated with the PhysioNet / CinC Challenge 2015; and (43, 60, 63) are dealing with SQIs and do not directly aim at arrhythmia alarms.

##### 3.3.6.1. SQIs for arrhythmia detection

SQIs are used for improving the detection of cardiac arrhythmia. Table 5 shows which SQIs are commonly used for which signals. A question mark (?) indicates that it is not clear from the publication alone if and how the respective SQI was used but this information can be inferred from context or references.

**Table 5:** SQIs used for arrhythmia detection.

Pub.	(104)	(105)	(106)	(107)	(108)	(109)
Aliases	“jSQI” aka. “SAI”	“ppgSQI”	“bSQI” and “kSQI”	“wSQI”	“sSQI”	
(78)	ABP	PPG				
(79)	ABP?	PPG?				
(80)		ABP+PPG	ECG			
(81)	ABP	PPG?				
(82)	ABP	PPG				
(84)	?			ABP + PPG		
(85)		PPG	?		?	
(88)	ABP	PPG				
(94)	ABP			ABP		
(61)	ABP					PPG

The table does not capture that for ECG signal quality assessment, custom methods are predominantly used. Eerikainen et al. (81) uses a combination of different signal quality measures, since (110) found that ECG SQIs differ in performance with varying types of arrhythmia. Eerikäinen et al. (66) is a follow-up publication to (81) and also uses a combinations of different signal quality measures on the ECG signal to build a custom SQI.

Xu et al. (85) state that they use a number of SQIs. However, they do not reference the SQIs in use only naming them as “iSQI,” “kSQI,” “ppgSQI,” and “sSQI” which are not referenced properly. For “ppgSQI” we can assume that (105) is the associated publication because it appears in the list references and the naming would be conclusive with the rest of the challenge. “kSQI” is referenced in (60) as (106) and “sSQI” is referenced in (60) as (108). For “iSQI” we did not find a corresponding reference. Sadr et al. (88) and He et al. (94) also use custom methods for assessing the ECG signal quality.

##### 3.3.6.2. Newly proposed SQI

Through our literature search we also found one publication proposing a novel SQI stating the alleviation of alarm fatigue as one possible purpose. Shahriari et al. (60) propose an image-based ECG SQI using the structural similarity measure (SSIM). The proposed SQI outperforms “baseSQI” (108), “kSQI” (106), and “sSQI” (108) which it was compared against.

##### 3.3.6.3. Review papers

Finally, there are two review papers on the available set of SQIs as well as their characteristics.

(43) focuses on signal quality assessments for ECG and ABP signals. However, since ABP and PPG are both pulsatile signals exhibiting a similar shape, approaches and findings for ABP signals are easily transferable to PPG signals. Concerning ECG quality assessment, the review’s main distinction is between time-domain and frequency-domain approaches as well as combinations of both.

Daluwatte et al. (63) assesses the effect of the beat detector on ECG SQIs. This is relevant because some SQIs require knowledge about the heartbeat positions in the ECG recording to make an assessment. This creates a circular problem because on the one hand, beat detectors are used to enable the signal quality assessment and on the other hand, signal quality assessments are used to judge the feasibility of a signal for beat detection.

## 4. Discussion

This literature review addressed two research questions: (1) What are IT-based approaches to improve alarm management in intensive-care medicine? And (2) how do these IT systems contribute to alarm management in intensive-care medicine? In the following, we summarise our findings for these research questions in an interleaved manner. For the IT systems we found, we present how these systems contribute to alarm management in terms of the data items defined in Section 2.6. Furthermore, we also summarise and discuss which kinds of systems we expected but did not find.

### 4.1. Summary of evidence and non-evidence

Alarm fatigue is a multicausal problem and approaches to alarm fatigue are multidisciplinary. This is confirmed by this review in a way that, even though we limited the domain of approaches to alarm fatigue to IT solutions, we still found a large variety of different works.

We found a diversity of objectives and approaches including assessment of the technical correctness of alarms, patient-specific risk, and alarm presentation. However, what is more interesting than what we found is what we expected but did not find as this manifests a need for future research. We did not find publications of systems classifying alarms according to their clinical relevance, i.e. whether an alarm is actionable or not. We suppose that this is due to a lack of data concerning this issue. Furthermore, there is a lack of systems affecting the timing of alarms. We found no systems for alarm forecasting and only one system proposing a strategical delay of alarms. Finally, we did not find systems that help to set reasonable alarm thresholds. Although there are heuristics in place in clinical practice for setting alarm thresholds, we did not find a system that can learn optimal alarm thresholds from data.

In terms of alarm types, we found that the majority of approaches targets heart rate or arrhythmia related alarms. This is reasonable since (11) found that these alarms represent the majority of all audible alarms.

The data item on hardware involvement showed that hardware is mainly used to introduce new means of alarm presentation or to facilitate simulations. There is a lack of publications using additional data sources such as inertial measurement unit (IMU) sensors which could be used for artefact detection and signal quality assessment which is currently done only via the signal itself (see Section 3.3.6).

Concerning data, the reviewed publications focus on custom data sets, MIMIC-II and the PhysioNet / Computing in Cardiology 2015 data set. More recent ICU databases like MIMIC-III, HiRID, and eICU CRD were not used which manifests a need for future research. Also, there is a need for ICU databases putting a stronger focus on alarms, hence facilitating alarm fatigue research.

Regarding the use of predictive models, there is a strong focus on rather simple and especially explainable models. Most publications either do not use models at all and rely on hand-crafted rules or use tree-based models. Complex models such as NNs, which are hard to explain and hard to investigate, are rarely used. We assume that this is to achieve acceptance by doctors and patients by showing the model’s decision-making process and thus avoid rare unanticipated effects which might cause harm in a clinical setting.

In our review, we found a wide range of different patient characteristics regarding age groups and specific conditions. It is, however, unclear how specific patient characteristics affect an IT solution. This is because every proposed IT solution was only evaluated with one set of patients and there are no comparative studies included in the scope of this review.

User studies were performed rarely and if so only with few (≤15) participants. This seems to indicate that most solutions – although promising – are far from being implemented as a regular part of clinical practice. Future work is needed to facilitate the adoption of new technologies to counteract alarm fatigue.

### 4.2. Limitations

There are a number of limitations relevant to this literature review.

#### 4.2.1. Scope

Alarm fatigue is a multimodal problem emerging not from a single source but from a variety of different aspects that need to be considered (1). This systematic literature review is limited to only include publications on computational approaches. There are, however, other domains to be considered as well, e.g. sound design and floor planning (10). We chose not to consider these other domains in this work because including all aspects of alarm fatigue in a single review would result in an unmanageable number of publications.

#### 4.2.2. Quantifiability

To the best of our knowledge, there is as of now no tool or metric available to quantify alarm burden. Therefore, we have no means to measure the effect each of the reviewed approaches has on the overall levels of alarm fatigue. Thus, we can only provide a qualitative summary of approaches aimed at alleviating alarm fatigue. Furthermore, even comparisons between very similar solutions are not always reasonable since the use of different data sets or otherwise changed circumstances might have a large impact on the efficacy of an approach.

### 4.3. Conclusions

We found that the IT-based solutions to alarm fatigue correspond to the nature of the alarm fatigue problem. The problem is complex and multicausal and hence the solutions address different causes and aspects of the problem, e.g. reduction of false alarms, alarm prioritisation, and alarm presentation. But we also found that existing research orients strongly on the existing data and not on clinical needs. Avoiding technically false alarms is a heavily researched topic because there are data available on this issue. However, there are few works on the avoidance of clinically irrelevant (i.e. non-actionable) alarms which might be due to the lack of available validated datasets on this issue.

Future research in this area needs to focus more on clinical needs and less on what is easily realisable from a technical perspective. Classifying alarms by clinical relevance (actionable or non-actionable) is an important next step. With an appropriate dataset of labelled alarms, machine learning models can perform this classification task. But datasets of relevance-labelled alarms do not exist as of yet or are at least not publicly available. An important next step in alarm fatigue research is creating such a dataset. In existing studies, clinical experts manually label alarms retrospectively, for example through inspecting video recordings of the situation. But manual labelling is time-consuming and thus limited to relatively few alarms. Another option is automatic annotation using a computer program that accesses the electronic health record and searches for interventions in response to the alarm. Automatic annotation can create a much larger dataset of labelled alarms and enables the creation of a machine learning classifier that helps clinical staff distinguish actionable from non-actionable alarms.

## Data Availability

The original contributions presented in the study are included in the article/Supplementary Material, further inquiries can be directed to the corresponding authors.

## Footnotes

^1^ https://dl.acm.org/

^2^ https://arxiv.org/

^3^ https://ieeexplore.ieee.org/Xplore/home.jsp

^4^ https://pubmed.ncbi.nlm.nih.gov/

^5^ https://www.webofknowledge.com

^6^ https://physionet.org/content/challenge-2015/1.0.0/

^7^ https://physionet.org/files/challenge-2015/1.0.0/papers/index.html
