# Supplementary figures and images for: Computational approaches to alleviate alarm fatigue in intensive care medicine: A systematic literature review
Source: Front Digit Health. 2022 Aug 16;4:843747. doi: 10.3389/fdgth.2022.843747 (PMC9424650; doi:10.3389/fdgth.2022.843747)

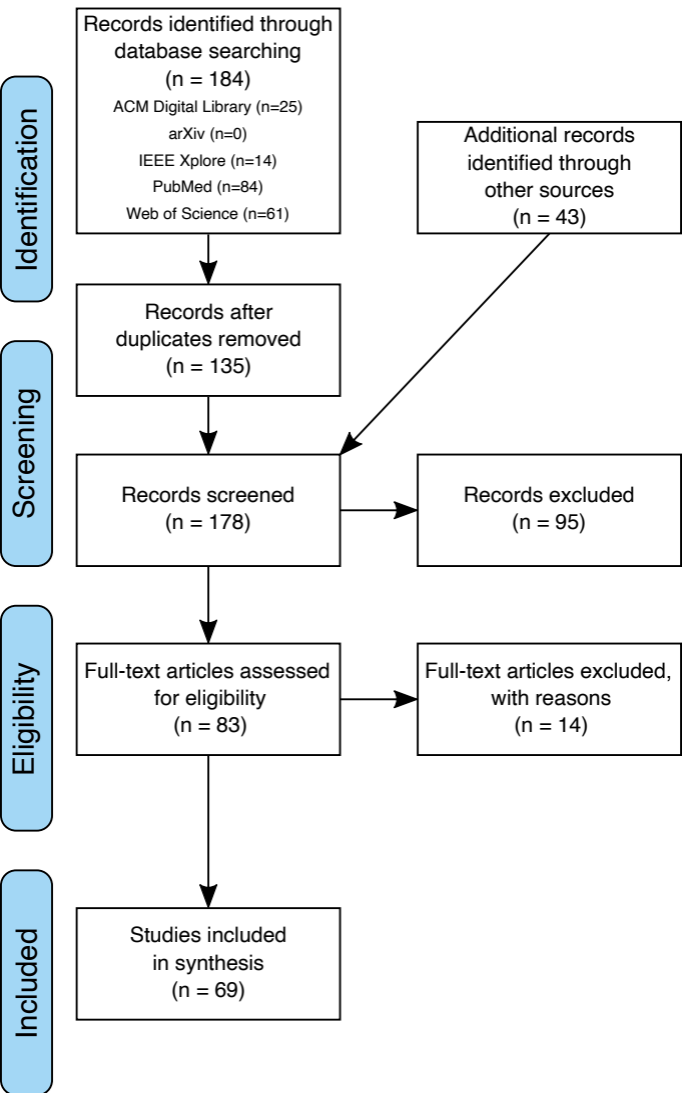

Supplement: Supplementary file 1 [file Data_Sheet_1_v1.pdf]
